# Brachialis Pulse Wave Measurements with Ultra-Wide Band and Continuous Wave Radar, Photoplethysmography and Ultrasonic Doppler Sensors

**DOI:** 10.3390/s21010165

**Published:** 2020-12-29

**Authors:** Horst Hellbrück, Gunther Ardelt, Philipp Wegerich, Hartmut Gehring

**Affiliations:** 1 Technische Hochschule Lübeck, University of Applied Sciences, 23562 Lübeck, Germany; gunther.ardelt@th-luebeck.de; 2 Institute of Biomedical Engineering, University of Lübeck, 23562 Lübeck, Germany; wegerich@imt.uni-luebeck.de; 3 Department of Anaesthesiology and Intensive Care Medicine, University Medical Center Schleswig-Holstein, 23538 Lübeck, Germany; hartmut.gehring@uni-luebeck.de

**Keywords:** pulse wave measurement, blood pressure, ultra-wideband, biomedical sensing

## Abstract

The measurement and analysis of the arterial pulse wave provides information about the state of vascular health. When measuring blood pressure according to Riva-Rocci, the systolic and diastolic blood pressure is measured non-invasively with an inflatable pressure cuff on the upper arm. Today’s blood pressure monitors analyze the pulse wave in reference to the rising or falling cuff pressure. With the help of additional pulse wave analysis, one can determine the pulse rate and the heart rate variability. In this paper, we investigated the concept, the construction, and the limitations of ultrawideband (UWB) radar and continuous wave (CW) radar, which provide continuous and non-invasive pulse wave measurements. We integrated the sensors into a complete measurement system. We measured the pulse wave of the cuff pressure, the radar sensor (both UWB and CW), the optical sensor, and ultrasonic Doppler as a reference. We discussed the results and the sensor characteristics. The main conclusion was that the resolution of the pulse radar was too low, even with a maximum bandwidth of 10 GHz, to measure pulse waves reliably. The continuous wave radar provides promising results for a phantom if adjusted properly with phase shifts and frequency. In the future, we intend to develop a CW radar solution with frequency adaption.

## 1. Introduction

In our project, the overall goal was to develop and validate a method for the non-invasive, continuous determination of arterial blood pressure. We chose a hybrid method with combined transmission and reflection measurements of ultra-wideband electromagnetic signals (ultra-wideband (UWB) and continuous wave (CW) radar) and ultrasonic signals (US) on the upper arm. In this study, we focus on the electromagnetic signals for pulse wave analysis. Today’s standard procedure for blood pressure measurement is according to Riva-Rocci—measure the systolic and diastolic blood pressure non-invasively with an inflatable pressure cuff on the upper right or left arm. The measurement and analysis of the arterial pulse wave provides additional information for medical application and diagnosis of the circulatory system. Recently, approaches for continuous non-invasive arterial pressure measurement (CNAP) [1] have evolved too.

**Figure 1:**
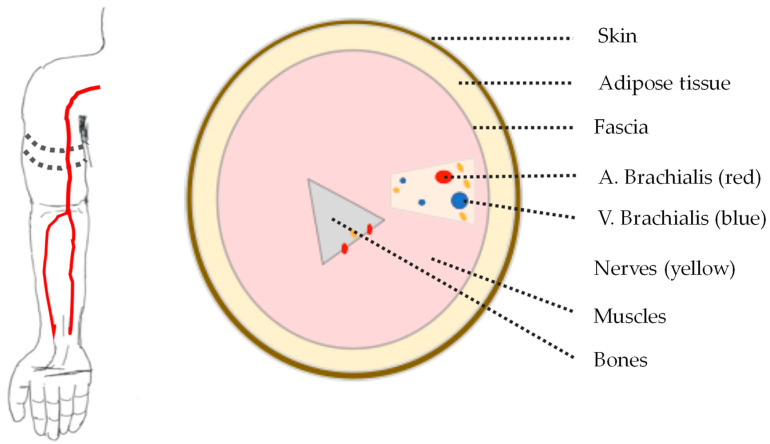
Illustration of a human arm with brachial artery (**left**) and schematic cross-section of upper arm (**right**).

According to J. Solà and R. Delgado-Gonzalo [2], pulse wave velocity (PWV) is directly related to blood pressure, and an accurate measurement of PWV delivers a measure of blood pressure if the relationship of PWV and blood pressure for a specific arterial path is given or known. Therefore, a measurement of pulse wave is a step forward in medical diagnostics, although an open problem in research is how to derive the blood pressure from PWV measurements. In this work, we aimed to investigate how far beyond the currently available methods such as ultrasonic approaches [3] can the measurement accuracy of a pulse wave can be increased by this novel approach with ultra-wide band electromagnetic signals. The aim of the development of the method was to focus on the upper arm region where the brachial artery runs through. The detection of low blood pressure should be possible as well as the parallel acquisition of the vessel wall extension and the flow velocity profile as seen in Figure 1.

Anatomically, the upper arm consists mainly of muscle tissue; humerus bone; fat tissue; skin tissue; and arterial, venous, lymphatic, and nerve plexus. The brachial artery runs along the inside of the upper arm and represents the artery typically used for blood pressure measurement with an upper arm cuff. It has an average inner vessel wall diameter of about 5 mm, which expands by up to 0.5 mm due to the pulsatile blood flow.

A setup of a sensor consisting of a transmitter and receiver pair with electromagnetic signals in the gigahertz range measure path length differences as echoes between the sensor and objects. Applied to a human body, the thorax movement of up to several millimeters are measured, which are caused by breathing or by heartbeat on the body surface, as shown in [4,5]. With the approach, non-invasive bio-parameters of individuals, such as respiratory rate and heart rate, can also be captured, as shown in [6,7]. The non-invasive measurement of the arterial pulse wave at arteries close to the surface was also performed contactless [8,9], as well as with sensor contact to the skin [10].

In principle, low-energy radar signals with frequencies of several gigahertz are suitable as sensor signals for measurement of the pulse wave (see [11]). Our major goal in this study was the measurement of the pulse wave and the cross-sectional area of the artery. In a future step, the arterial measurements will be included in the calculation of the blood pressure to improve the blood pressure measurements, as shown in [12,13,14]. For this purpose, ultrasonic sensor arrays are suitable as they provide a depth-selective contrast image of the tissue composition from reflection signals, from which the cross-sectional area of the vessel can be extracted. However, the blood pressure cannot be determined in this way; calibration is necessary. Non-invasive blood pressure measurement is still performed with an upper arm pressure cuff. Blood pressure can be determined accurately, but not continuously. The cuff measurement is indispensable as a reference measurement of blood pressure, and it serves as a calibration value for indirect methods of blood pressure determination such as pulse wave analysis. Suitable UWB and US signals should be analyzed and selected to measure vessel wall expansion and flow velocity profile. A sensor system should be designed and implemented as a prototype in an upper arm cuff. Algorithms for the calculation of blood pressure should be developed in future work for standardized testing of the system on the model and on the test person.

The structure of the paper is as follows: Section 2 introduces the materials and methods, where we describe the model environment and the signals, as well as processing for the RF radar technology. In Section 3, we present the measurement setup, the system and measurement architecture, and the results, with preliminary discussions where we perform measurements with two variants and to test setups. Section 4 discusses the results comparatively and Section 5 concludes the paper.

## 2. Materials and Methods

The tasks are divided into the development of a model environment for the evaluation of the process, the development of the process, and the development of algorithms for feature extraction. For this purpose, the following tasks were accomplished.

First, explicit knowledge about UWB signals, the anatomy of the upper arm, and electromagnetic tissue properties of the upper arm was researched and summarized. Then UWB signals were simulated and examined practically with the existing high-frequency technology. In the next step, suitable UWB antennas were purchased to evaluate the transmission properties of UWB signals for the application. Additionally, simulations of the antenna properties in the frequency domain were performed. A model environment mapped the anatomical and physiological properties of the upper arm onto a laboratory model in the form of a phantom. The phantom was supplemented with corresponding components for the generation of pressure and flow profiles.

Parallel to this, various UWB systems were prototypically built, which can be integrated into the phantom and are also suitable for measurements on the upper arm. This served the development of the method. An application was approved by the ethics committee of the University of Applied Sciences Lübeck in order to be able to test the prototypical systems functionally on the upper arm. The hybrid approach with UWB was prepared and supplemented with optical sensors, pressure sensors, and US flow sensors, which serve as a reference.

Algorithms were developed on the basis of the measured UWB signals to determine changes in vessel wall diameter in the model as a feature. The extracted features were compared with the reference data.

### 2.1. Development of a Model Environment for Evaluation

Research on UWB signals shows that broadband single pulses as well as sequences of these pulses are suited for the application. The more broadband the UWB signal is in the frequency domain, the narrower the pulse is in the time domain, and the higher the resolution. In the targeted frequency range of 3–6 GHz the wavelength is about 5–10 cm in vacuum. In lossy media, the wavelength decreases. In tissue, a reduction of the wavelength by a factor of 2 to 7 is expected, depending on its permittivity. The reason behind this is that the speed of electromagnetic signals in tissue is reduced by this factor. The temporal extension of a pulse is typically about 1 ns. During this time, the pulse travels a distance of about 30 cm in a vacuum, and thus the distance in tissue is correspondingly shorter.

Certain pulse sequences, which can be used as UWB signals, have advantageous correlation properties in the time domain. These sequences are known as pseudobinary random sequences. They correlate with themselves only at a single point in time and enable accurate distance measurement to a target object. The shortest sequences have a length (pulse count) of 7 (see [15,16]). Furthermore, with pseudobinary random sequences, multiple echoes can be distinguished.

The penetration of UWB pulses into tissue and artery was investigated in a time domain simulation. The simulation depicts the model environment upper arm with the medium NaCl solution in different concentrations to simplify the contrast between tissue and blood. The brachial artery is modeled as a cylindrical tube. The simulation was programmed in COMSOL Multiphysics. The UWB pulse shape is arbitrarily malleable and the geometry of the model environment is freely parameterizable. In the simulation, a UWB pulse was radiated into the tissue model. A Vivaldi antenna, which is surrounded by vacuum, is in contact with the upper arm, represented as a material with a conductivity of 1 S/m and a relative permittivity of 22. The brachialis is 10 mm in diameter, with a wall thickness of 0 mm, modeled with a conductivity of 2 S/m and a relative permittivity of 78. The brachialis is 20 mm apart from the contact area of the antenna and the arm tissue. The simulation results revealed that no information about the vessel wall diameter can be extracted from pulses themselves as the resolution was by several magnitudes lower than the change in the wall diameter.

**Figure 2:**
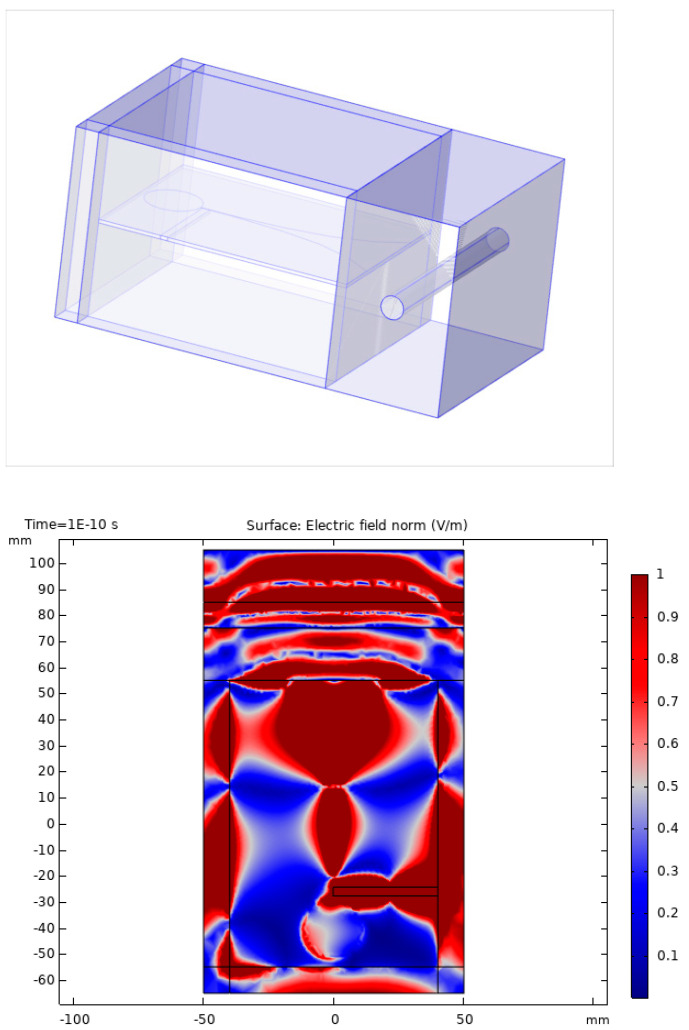
COMSOL simulation of microwave electromagnetic signals and reflection of the waves at borders in a macro scale. The left part is the antenna, and the right part shows the simple model with the artery in a salty water environment.

Figure 2 illustrates a snapshot of the simulation with COMSOL Multiphysics [17,18] in time domain performed for evaluation in our project. The signal was emitted as a short pulse by the antenna placed at the left side of the topology. The tissue and the artery were placed at the right side. The color indicates the signal strength of an emitted electromagnetic pulse after first reflections and 100ps. The simulations showed that several reflections interfered and that the wavelength of several centimeters did not allow for identification of individual reflections.

**Figure 3:**
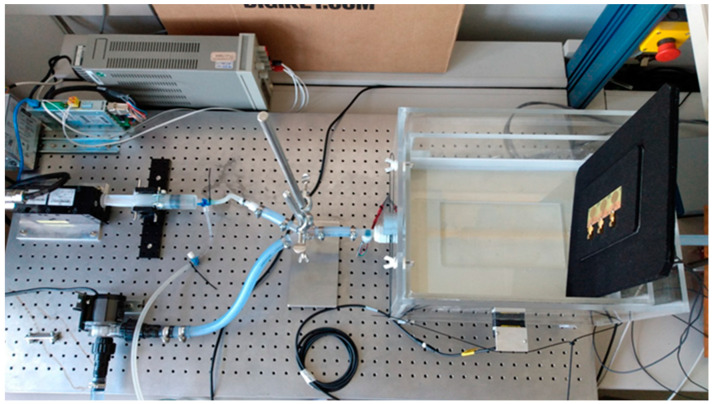
Setup for macroscopic model environment for measurement setup.

The model environment (phantom) illustrated in Figure 3 was designed and implemented, which reproducibly simulated a variable vessel expansion and a variable flow. The parameters were the same as for the simulations in Figure 2. The complex anatomical, physiological, electromagnetic, and sonographic properties of the upper arm were simplified. Different dynamic vascular states can be realized by the setup.

The model environment was built up modularly. The main components are a tissue phantom, a pump to generate a pulsatile flow or pressure, and a pump to generate a constant flow or pressure. The tissue phantom reproduced the characteristics of the brachialis in the form of a latex tube. The upper arm tissue was modeled with NaCl solution, a blood replacement within the tube system via different NaCl concentration. To simulate the arterial pulse wave, we built up a linear motor with a piston syringe to a piston pump. The pulse shape and pulse sequence that the piston pump outputs were configured by were the parameters output volume, rise time, and repetition frequency. With the additional gear pump, a constant flow and pressure in the phantom artery can be adjusted. The pressure is continuously measured invasively at the phantom artery inlet and outlet using pressure sensors in contact with the media. For the insertion of UWB and US sensors, the phantom has a measuring window that was separated from the phantom tissue only by a thin latex foil. The distance of the measuring window to the phantom artery is continuously adjustable.

### 2.2. Development of the Signal Processing

The UWB antennas have a frequency range from 3 to 6 GHz. They have an omnidirectional radiation pattern and geometric dimensions of half a wavelength in the corresponding frequency range. The antenna has a constant group delay in a wide frequency range, which is important for the transmission of broadband signals. A finite element model (FEM) simulation of the UWB antenna in the frequency domain shows qualitatively the specified manufacturer behavior. Pulses with a bandwidth of up to 200 MHz can be transmitted nearly distortion-free over the whole frequency range.

The further signal chain includes the processing of the antenna signal by power and it receives amplifiers and filters. On the source side, all signals are generated by a precision high-frequency (HF) arbitrary generator. Received signals are recorded segmented by an oscilloscope and digitally processed or digitized by analog mixers in the baseband. There are system technical limitations due to the memory of the oscilloscope, whose volume limits the measuring time to approximately 100 milliseconds. Thus, a high bandwidth is possible, but there is no observation over a relevant period of time with respect to the bandwidth and duration of an arterial pulse wave. On the other hand, the analog mixer is narrow band, with a total bandwidth of 20 MHz, and thus pulse shapes cannot be transmitted. Therefore, a relevant time period with high bandwidth can be observed dynamically, but the dimension of the target site is lost. With these measurement concepts, we developed two prototype systems. The first system is a UWB pulse radar, and the second system is a continuous mono-frequency (CW) radar.

With the pulse radar, any pulses and pulse sequences in the frequency range 3 to 6 GHz can be transmitted. The time resolution is increased by a factor of 10 down to a resolution of 1 ps by subsequent interpolation, which corresponds to a sampling rate of 1 THz. By measuring the pulse propagation time, a distance resolution of about 0.3 mm is achieved. The distance resolution is much higher with a shorter pulse or increased sequence length.

**Figure 4:**
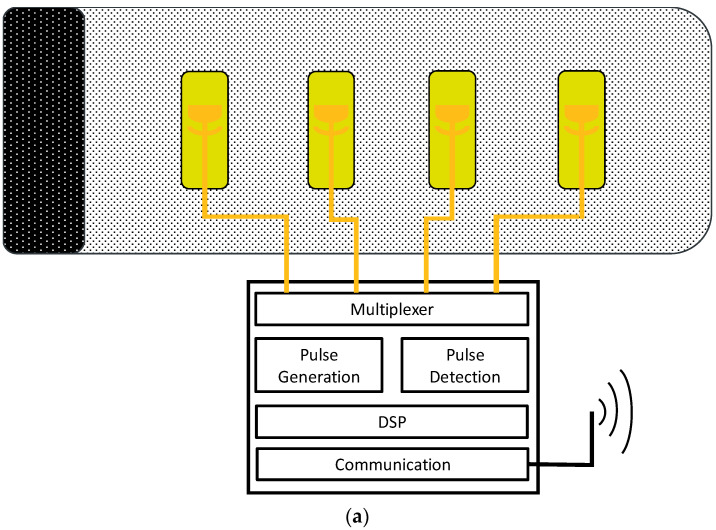

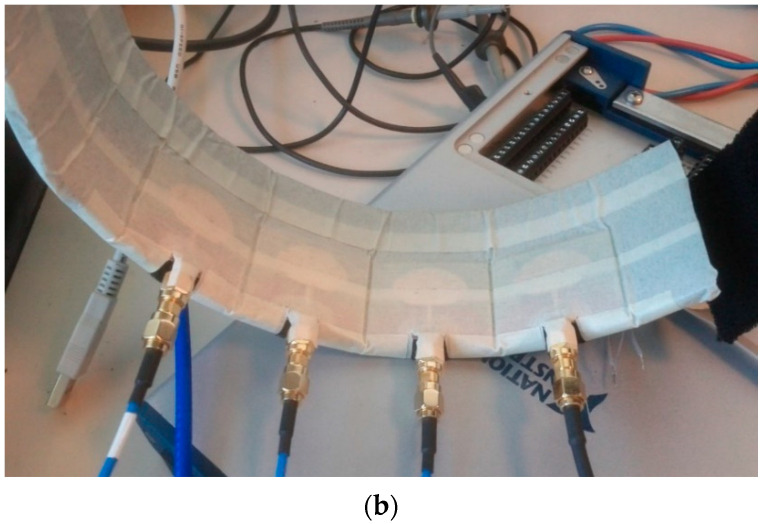
Concept and implementation of sensor array for application at the upper arm to measure pulse wave. The electrical setup consists of four antennas connected to a multiplexer with a pulse generator and detector with digital signal processor (DSP) and a wireless communication interface (**a**). The antenna setup and cabling for a prototype (**b**) is to be wrapped around the upper arm.

**Figure 5:**
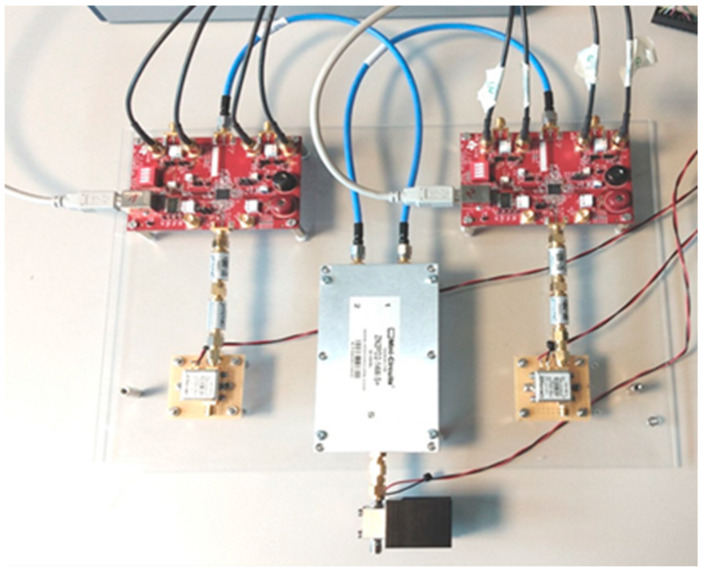
Electronic components with two separate receiver paths including I/Q demodulation on the right side and the left side. Receiver antennas are not connected to the low noise amplifier (LNA) at the bottom (left and right). In the middle, we used a splitter for a 500 to 10,500 MHz frequency range for the two receiving paths. Carrier signal is inserted at the bottom of the setup.

The CW radar can be operated in the same frequency range. Due to the analog-direct mixing, this is not necessary in the digital part of the signal processing. Thus, the interferometric superposition of the received signal and the transmitted signal can be observed continuously and in real time. A resolution of about 0.02 mm can be realized with a bandwidth of 1 kHz. Both radars can be used as modular arrays. The pulse radar can be operated sequentially with up to 4 transmit and receive antennas each, which are arranged as a ring or area array. The CW radar can be operated with two transmit (TX) and receive (RX) antennas in parallel, as shown in Figure 4 and Figure 5. Figure 4a illustrates the schematic view. Figure 4b is a photo of the antenna array. The receiver electronics in Figure 5 consist of two receiver paths and a synchronous I/Q demodulation.

## 3. Results

The first basic tests for evaluation of the setup of the pulse radar in open air confirmed that moving targets in the far field of the antenna can be detected reliably, as given in [19]. This was revealed with the correlation of transmit pulse and receive pulse in the time domain, as well as with a Doppler evaluation, which returns target location and frequency shift. In the near field of the antenna, the temporal correlation was no longer possible, because the transmitting pulse was so far extended in time that a receiving pulse disturbed by this was lost. However, this reflected the later application case where the artery was in the near field. Additionally, the required distance resolution could not be achieved this way. This would require much higher signal frequencies, which in turn would not penetrate the tissue. In principle, the CW radar test showed a high distance resolution. This was achieved by measuring the dynamic phase shift when the target was moving. A prerequisite for this is a defined phase position between transmitter and receiver, which must be adjusted beforehand. However, this also depends on the environment of the antennas, and thus an unnoticed change in the antenna-environment arrangement will unintentionally detune the interferometer.

**Figure 6:**
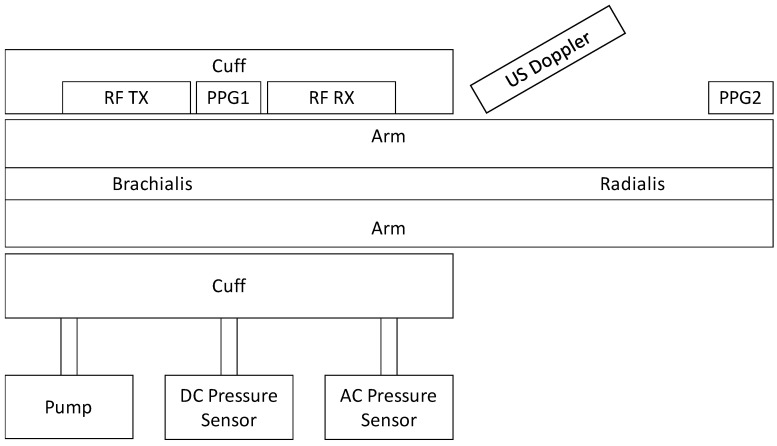
Complete measurement setup for blood pressure and pulse wave measurements for comparison and evaluation of ultrasonic, ultra-wide band radio frequency (RF) and optical signals.

The hybrid approach with integrated US was implemented with a commercial, existing CW-US Doppler system, as given in Figure 6. The US system consisted of a transducer operating at a signal frequency of 5 MHz. The transducer was a point source that was applied at a defined angle to the target and could be flexibly integrated in close proximity to the UWB system. The transducer was in direct skin contact with an ultrasonic gel. Synchronicity between the CW radar and US Doppler was ensured by a multi-channel analog-to-digital conversion, which sampled all incoming signals of the different systems with the same sample rate of 48 kHz. The US Doppler had been modified in order to output analog I/Q-demodulated raw signal of the transducer.

To increase the accuracy of the measurement, we implemented two synchronous CW radars with identical signal processing. However, note that antennas were separated in space and were in direct contact with the skin that was only covered with crepe paper tape, as shown in Figure 4. The antennas were not yet adopted to the use case to transmit and receive signals to and from a human body. We will optimize the antennas in the future.

Measurements in this setup were performed with individual humans sitting on an office chair and breathing normally without moving too much. In this paper, we showed a typical result of these measurements from one test person. Additional tests were performed with two more healthy male individuals between 30 and 40 years old, providing similar results. Similar means that we were able to achieve a similar strength (amplitude) and shape of the signal between the individuals. Moreover, reproducibility was given when measurements were repeated. However, the results depended very much on the location, orientation, and the pressure to the skin of the cuff. A variation of some millimeters would affect the results in an unpredictable way, and thus in the worst case the signal would disappear in the noise floor with a very low amplitude. Therefore, in practical experiments, we need to tune the location, orientation, and pressure of the antenna to the skin to again achieve a strong and good signal for each test person.

**Figure 7:**
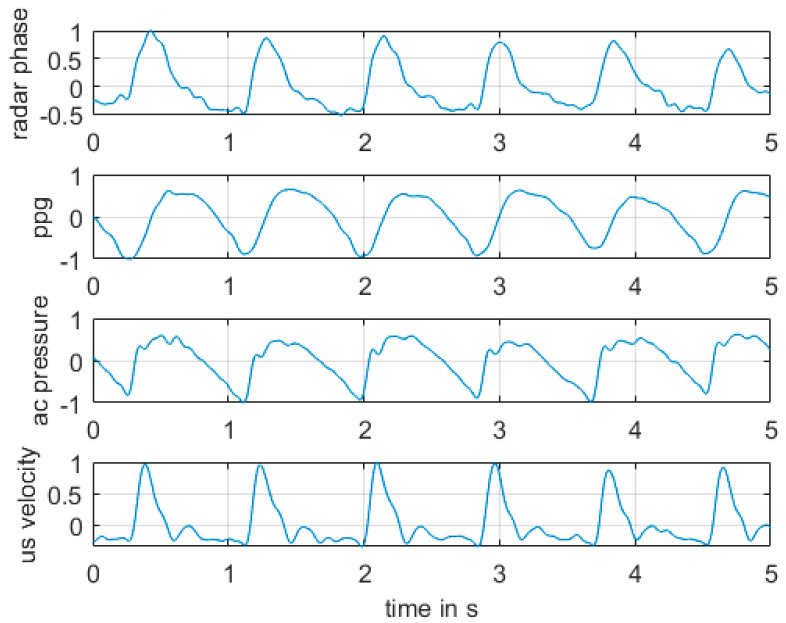
Results for measurement according to Figure 6. All shown measurement signals are normalized to 1. From top to bottom: phase between radar signals I and Q; photoplethysmographic signal 1; ac pressure signal in cuff; ultrasound velocity signal. Bandpass filter Chebyshev type 1 with order 6 infinite impulse response and passband 0.5 Hz–10 Hz was applied to all signals.

Figure 7 shows the result of measurements for pulse wave radar at the upper arm of a human in comparison with PPG, AC-pressure, and US Doppler. All signals were bandpass filtered with the same parameters. See details of the parameters in the caption of Figure 7.

We found similarities for all curves showing the pulses of the blood circularity system. The ultrasonic sound and the radar phase had very similar shape and required further investigation in terms of their major similarities. We do not yet know if the phase signal from the CW radar was from the change of the diameter or the movement of the blood from these experiments. However, the results for the pulse wave radar were not stable. A small movement or change of the contact pressure of the antenna system can change the resulting measurement signal in an unpredictable way, as already discussed in the previous paragraph describing the setup of the measurements. Therefore, we did not perform a statistical evaluation of the measurements. For this reason, we investigated the continuous wave radar in more detail and in a systematic way in a macroscopic model environment in the next step.

### Algorithms for Feature Extraction

The UWB system was integrated into the Phantom as a CW radar and was in contact with the outer latex foil. The CW-US Doppler system was statically mounted at the phantom artery outlet. The transmitted signal power can be observed by bypass measurements on a standard oscilloscope and controlled by the arbitrary waveform signal generator. For precise measurement of the diameter of the phantom artery, we integrated an optical distance sensor into the model environment, which measured the wall movements in high resolution. All sensor values—CW radar, US Doppler, pressure, and optical distance sensor—were sampled and stored synchronously and automatically by the multi-channel data acquisition card.

**Figure 8:**
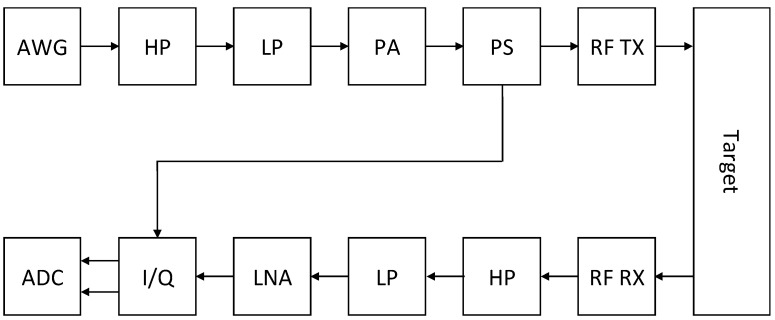
Block diagram of transmitter and receiver path, including generation and demodulation for abbreviations (see Table 2).

Figure 8 shows the processing chain from the signal generation to the raw digital measurement results. The signal was generated by an arbitrary waveform generator (AWG). A high and low pass filter (HP7LP) suppressed unwanted signal components from the input signal. The power amplifier (PA) amplified the signal ready to be applied to the RF transmission antenna. A power splitter (PS) generated a copy of the signal for synchronization with the received signal from the receiving antenna. The receiving path started with the RF receiving antenna and a low noise amplifier (LNA). Unwanted signal components were filtered by high and low pass filter before demodulation with an I/Q demodulator fed with the phase shifted transmitted signal. A dual channel analog to digital converter created digital I and Q signals from the analog sources. These raw signals were processed further. The phase angle between I and Q was measured and the signals were filtered again, e.g., low pass filter to create the pulse wave measurement. 

**Figure 9:**
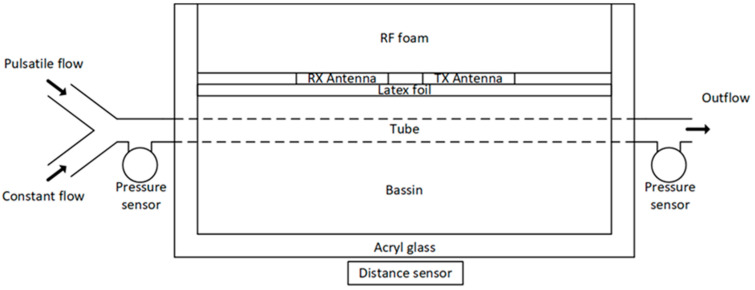
Macroscopic model environment for evaluation of continuous wave radar with pressure and distance reference measurements.

The macroscopic model as illustrated in Figure 9 consists of an acryl glass basin filled with water, where an elastic latex tube with an outer diameter of 14mm. The antennas are separated from the water by a latex foil. The conductivity of the water in the water hose system flowing through the tube is 4 times higher (3.2 S/m). All mechanical and electrical parameters are given in Table 1.

Parameterized measurements with imprinted pressure and flow profiles show that a vessel wall expansion can be measured reproducibly. These measurements can be carried out with variable positioning of the antennas. Different signal filters were tested. Suitable filters were Chebychev infinite impulse response (IIR) filters of type 1 and 2. An additional forward and backward filtering in the time domain ensured the phase-true signal filtering. All sensor signals could be analyzed in time domain and frequency domain with flexible sample rate and bandwidth. For extraction of the vessel diameter in the model environment, we observed the phase shift of the CW radar signal as a function of frequency and medium. The phase shift was converted into a distance change between radar antenna and phantom artery with the wavelength according to

$$\phi_{m}\left[\mathrm{mm}\right]=\phi_{iq}\lambda \left(f\right)$$

The measured distance change of the optical sensor served as reference and calibration. Under otherwise identical conditions of the model environment, the values of the different sensors correlated.

**Figure 10:**
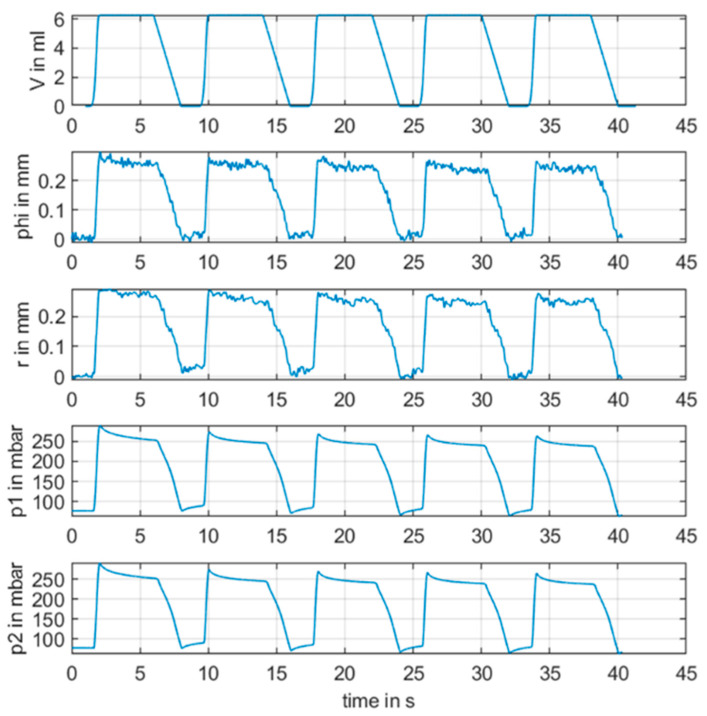
Comparison of measurements from all sensors. Phi is $\phi_{m}\left[\mathrm{mm}\right]$ from RF continuous wave radar measurements (CW) with a frequency of 4.1 GHz, *p1* and *p2* are the pressure sensors, *r* is the distance sensor, and *V* is the injected volume over time.

**Figure 11:**
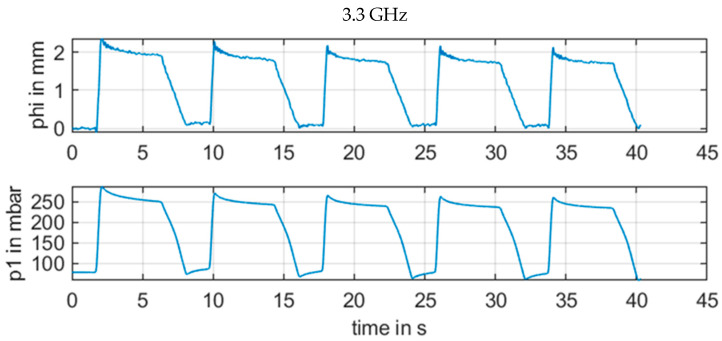
Measurement results for $\phi_{m}\left[\mathrm{mm}\right]$ of CW radar with frequency of 3.3 GHz in comparison with reference pressure *p1* from pressure sensor.

**Figure 12:**
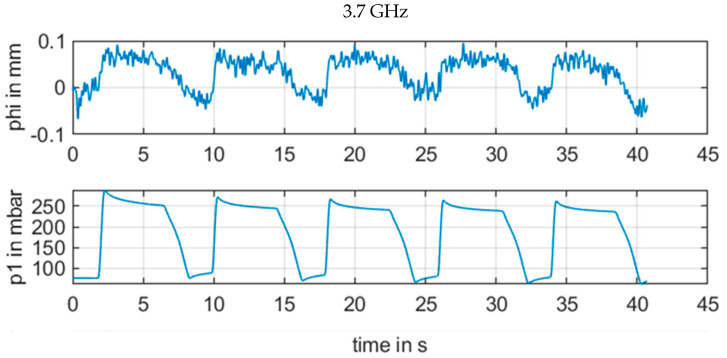
Measurement results for $\phi_{m}\left[\mathrm{mm}\right]$ of CW radar with frequency 3.7 GHz in comparison with reference pressure *p1* from pressure sensor.

**Figure 13:**
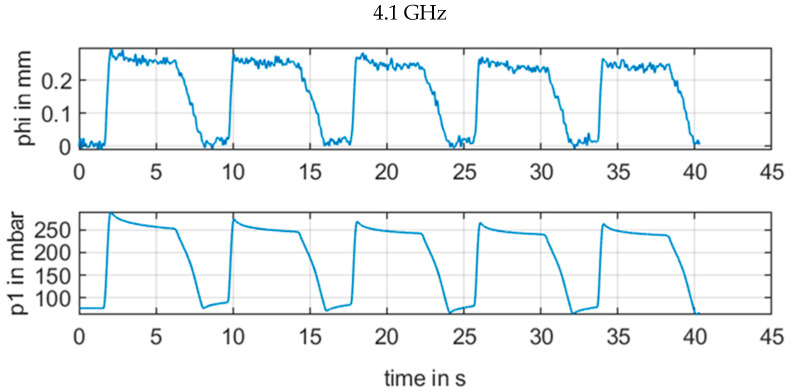
Measurement results for $\phi_{m}\left[\mathrm{mm}\right]$ of CW radar with frequency 4.1 GHz in comparison with reference pressure *p1* from pressure sensor.

**Figure 14:**
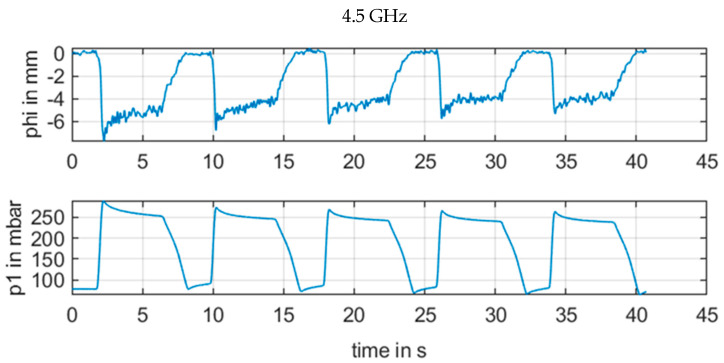
Measurement results for $\phi_{m}\left[\mathrm{mm}\right]$ of CW radar with frequency 4.5 GHz in comparison with reference pressure *p1* from pressure sensor.

We performed measurements with injection of a liquid volume with a repetitive a pulse and a volume of 6 mL for a duration of 6 s and a pause of 2 s. Every 8 s, we repeated the pulse four times. The radar continuous frequencies were 3.3 GHz, 3.7 GHz, 4.1 GHz, and 4.5 GHz for the CW radar. Values from CW were low-pass filtered up to 5 Hz without attenuation. The low pass filter had an attenuation of 60 dB for 30 Hz. Results of this setup are shown in Figure 10 and Figure 11, Figure 12, Figure 13 and Figure 14 with the different frequencies from 3.3 to 4.5 GHz.

Figure 10 illustrates the results of the measurements. There was a strong correlation between the input signal volume *V* and measured signals from all sensors. It seemed as though CW radar provided the same stable measurement values as the other sensors.

However, a change in the frequency in Figure 11, Figure 12, Figure 13 and Figure 14 revealed how susceptible the radar measurements were for small changes in the setup in contrast to the other sensors. Figure 11 with 3.3 GHz showed a large signal amplitude for CW radar, whereas Figure 12 with 3.7 GHz demonstrated a low amplitude noisy measurement signal. In Figure 13 at 4.1 GHz, the signal was again stronger, and finally in Figure 14, with a CW frequency of 4.5 GHz, the signal was inverted.

## 4. Discussion

In this paper, we implemented and evaluated a (a) ultra-wide band (UWB) pulse radar and (b) continuous wave (CW) radar for pulse wave measurements to improve blood pressure measurement. The goal was to develop a new continuous non-invasive reliable measurement standard in medicine. Our results confirmed that RF measurements have some advantages and disadvantages. One of the main drawbacks of RF signals is that the resulting wavelength is much larger in comparison to ultra-sonic signals due to the much higher propagation speed and the limited maximum frequency for electromagnetic signals, in accordance with the work in [20]. Electromagnetic signals with more than 10 GHz experience a high attenuation in adipose and muscle tissue according to Bilich [20].

For ultra-wide band (UWB) pulse radar (a), the limitations are the pulse length in time and in distance that are also in the range of several centimeters. Therefore, pulses overlap too much to be separated and processed at the upper arm with all reflections. Figure 2 illustrates the problem where a short pulse was transmitted, and resulting multiple echoes were difficult to separate from each other.

For continuous wave (CW) radar (b), the wavelength in the range of several centimeters also resulted in a strong and unpredictable interference between electromagnetic waves from various reflections for continuous wave signals with minor phase shifts of less than λ/4. For the measurement situation at the upper arm, there were several reflections at the artery brachialis, at bone material, veins, and skin, etc. This resulted in an unpredictable phase signal, as illustrated in Figure 11, Figure 12, Figure 13 and Figure 14. The signal quality varied with a moderate variation of the RF frequency of the CW radar, which is a key parameter to produce a strong measurement signal

In summary, the new method is not yet ready to be applied in real situations and its usability in clinical practice is not yet given. The feasibility of the technique needs to be improved to reduce the complexity of the handling of the measurement system before being applied in clinical practice.

For the future, we need an improved model of the upper arm, especially for electromagnetic characteristics and for including the dynamics of the pulse wave, so that the phase shifts of the CW radar can both be interpreted better and be mapped to changes in the diameter of the artery. This includes antenna design with optimized radiation pattern, which has not yet been addressed. A basis for this optimization is the work from Jacobi et al. in [21] or [22,23]. A second direction is the adaptation of the frequency to achieve a high signal quality in terms of stability and signal-to-noise ratio. One candidate for further investigation and development is the radar-on-chip solution, as introduced recently by Lauteslager et al. in [24]. Compared to their investigations, we already have a macroscopic model environment with integrated pressure and US Doppler sensors for evaluation of UWB signals.

## 5. Conclusions

In this paper, we described radio frequency systems with pulse radar and continuous wave radar and discussed their limitations for pulse wave measurements of the brachial artery at the level of the upper arm. The result was that none of the two technologies were able to solve the problem in their basic setup with a stable reliable measurement. The resolution of the pulse radar was too low, even with a maximum bandwidth of 10 GHz to measure pulse waves reliably. The continuous wave radar provided promising results for a phantom if adjusted properly with phase shifts and frequency. When applied to a real human arteria brachialis on the upper arm, the setup was so dynamic that even the smallest movement of the arm or the antennas had unpredictable effects. Nevertheless, RF systems have many advantages for non-invasive measurements. Therefore, the next step is a self-stabilizing system that applies continuous wave signal with adjusted frequencies for a reliable strong output signal. An improvement is expected by the use of coherent ultra-wide band radar technologies.

## Figures and Tables

**Table 1:** Macroscopic model parameters.

Parameter	Value and Unit
Length latex tube	320 mm
Inner diameter of latex tube	10 mm
Outer diameter latex tube	14 mm
Pretension latex hose	10 Nm
Horizontal center distance between antennas	42 mm
Vertical distance between antennas and latex tube	8 mm
Water basin conductivity	0.8 S/m
Water hose system conductivity	3.2 S/m

**Table 2:** Abbreviations for Figure 8.

Abbreviation	Explanation
AWG	Arbitrary waveform generator
HP	High pass filter
LP	Low pass filter
PA	Power amplifier
PS	Power splitter
RF TX	RF transmitting antenna
RF RX	RF receiving antenna
LNA	Low noise amplifier
I/Q	IQ demodulation
ADC	Analog to digital conversion

## Data Availability

The data presented in this study are available on request from the corresponding author. The data are not publicly available due to technical reasons.

## References

[1] Jeleazcov C., Krajinovic L., Münster T., Birkholz T., Fried R., Schüttler J., Fechner J. (2010). Precision and accuracy of a new device (CNAP^TM^) for continuous non-invasive arterial pressure monitoring: Assessment during general anaesthesia. Br. J. Anaesth..

[2] Solà J., Delgado R. (2019). The Handbook of Cuffless Blood Pressure Monitoring: A Practical Guide for Clinicians, Researchers, and Engineers.

[3] Beulen B.W., Bijnens N., Koutsouridis G.G., Brands P.J., Rutten M.C., Vosse F.N. (2011). Toward noninvasive blood pressure assessment in arteries by using ultrasound. Ultrasound Med. Biol..

[4] Bi S., Liu X., Matthews D. An experimental study of 2-D cardiac motion pattern based on contact radar measurement. Proceedings of the 2015 IEEE 16th Annual Wireless and Microwave Technology Conference (WAMICON).

[5] Bi S., Zeng J., Bekbalanova M., Matthews D., Liu X.L. Contact-based radar measurement of cardiac motion—A position and polarization study. Proceedings of the 2016 IEEE Topical Conference on Biomedical Wireless Technologies, Networks, and Sensing Systems (BioWireleSS).

[6] Immoreev I., Tao T.-H. (2008). UWB radar for patient monitoring. IEEE Aerosp. Electron. Syst. Mag..

[7] Immoreev I.Y. Practical Application of Ultra-Wideband Radars. Proceedings of the 2006 3rd International Conference on Ultrawideband and Ultrashort Impulse Signals.

[8] Kim I., Bhagat Y.A. Towards development of a mobile RF Doppler sensor for continuous heart rate variability and blood pressure monitoring. Proceedings of the 2016 38th Annual International Conference of the IEEE Engineering in Medicine and Biology Society (EMBC).

[9] Shi K., Schellenberger S., Steigleder T., Michler F., Lurz F., Weigel R., Koelpin A. Contactless Carotid Pulse Measurement Using Continuous Wave Radar. Proceedings of the 2018 Asia-Pacific Microwave Conference (APMC).

[10] Ebrahim M.P., Heydari F., Wu T., Walker K., Joe K., Redoute J.-M., Yuce M.R. (2019). Blood pressure estimation Using on-body continuous Wave Radar and photoplethysmogram in Various posture and exercise conditions. Sci. Rep..

[11] Barnes F.S. (2018). Biological and Medical Aspects of Electromagnetic Fields.

[12] Gesche H., Grosskurth D., Küchler G., Patzak A. (2012). Continuous blood pressure measurement by using the pulse transit time: Comparison to a cuff-based method. Eur. J. Appl. Physiol..

[13] Hirata K., Kawakami M., Rourke M.F. (2006). Pulse Wave Analysis and Pulse Wave Velocity. Circ. J..

[14] Sugawara M., Niki K., Furuhata H., Ohnishi S., Suzuki S. (2000). Relationship between the pressure and diameter of the carotid artery in humans. Heart Vessel..

[15] Addad M., Djebbari A. (2017). A ternary zero-correlation zone sequence sets construction procedure. Turk. J. Electr. Eng. Comput. Sci..

[16] Funahashi Y., Nakamura K. (1974). Parameter estimation of discrete-time systems using short-periodic pseudo-random sequences. Int. J. Control.

[17] Midasala V., Siddaiah P., Bhavanam S. Design of dipole antenna by using COMSOL Multiphysics Software. Proceedings of the International Conference on Electronics and Communications Engineering (ICECE 2014).

[18] Wiwatwithaya S., Phasukkit P., Tungjitkusolmun S., Wongtrairat W. Real-time monitoring glucose by used microwave antenna apply to biosensor. Proceedings of the 4th 2011 Biomedical Engineering International Conference.

[19] Alabaster C. (2012). Pulse Doppler Radar: Principles, Technology, Applications.

[20] Bilich C.G. Bio-medical sensing using Ultra Wideband communications and radar technology: A feasibility study. Proceedings of the 2006 Pervasive Health Conference and Workshops, Pervasive Health.

[21] Jacobi J.H., Larsen L.E., Hast C.T. (1979). Water-immersed microwave antennas and their application to microwave interrogation of biological targets. IEEE Trans. Microw. Theory Tech..

[22] Liang J., Chiau C.C., Chen X., Parini C.G. (2005). Study of a printed circular disc monopole antenna for UWB systems. IEEE Trans. Antennas Propag..

[23] Liang X.L., Matin M. (2012). Ultra-wideband antenna and design. Ultra Wideband—Current Status and Future Trends.

[24] Lauteslager T., Tømmer M., Lande T.S., Constandinou T.G. (2019). Coherent uwb radar-on-chip for in-body measurement of cardiovascular dynamics. IEEE Trans. Biomed. Circuits Syst..

